# A metastasis‐associated microRNA‐based liquid biopsy signature for risk‐stratification in colorectal cancer: a multicenter cohort study

**DOI:** 10.1002/ctm2.998

**Published:** 2022-12-13

**Authors:** Takatoshi Matsuyama, Yuji Toiyama, Toshiaki Ishikawa, Yoshinaga Okugawa, Masamichi Yasuno, Joan Maurel, Yusuke Kinugasa, Hiroyuki Uetake, Ajay Goel

**Affiliations:** ^1^ Center for Gastrointestinal Research, Center for Translational Genomics and Oncology, Baylor Scott & White Research Institute and Charles A Sammons Cancer Center Baylor University Medical Center Dallas Texas USA; ^2^ Department of Gastrointestinal Surgery Tokyo Medical and Dental University Graduate School of Medicine Tokyo Japan; ^3^ Department of Gastrointestinal and Pediatric Surgery, Division of Reparative Medicine, Institute of Life Sciences Graduate School of Medicine, Mie University Mie Japan; ^4^ Department of Specialized Surgery Tokyo Medical and Dental University Graduate School of Medicine Tokyo Japan; ^5^ Translational Genomics and Targeted Therapeutics in Solid Tumors Group Medical Oncology Hospital Clinic of Barcelona, CIBERehd, IDIBAPS Barcelona Spain; ^6^ Department of Molecular Diagnostics and Experimental Therapeutics Beckman Research Institute of City of Hope Monrovia California USA; ^7^ City of Hope Comprehensive Cancer Center Duarte California USA

**Abbreviations**
AUC: area under the curve
CI: confidence interval
CRC: colorectal cancer
DFS: disease‐free survival
HR: hazard ratio
miRNA: microRNA
ROC: receiver operating characteristic

Dear Editor,

Colorectal cancer (CRC) continuously sheds various subcellular components into the bloodstream, including microRNAs (miRNAs).^1^, ^2^, ^3^ In the current study, we systematically and comprehensively profiled miRNAs in patients with CRC to test the hypothesis that miRNAs highly expressed in metastases are shed into the bloodstream and can act as promising blood‐based biomarkers to aid in clinical decision‐making, which led us to identify clinically translatable circulating miRNAs for recurrence prediction in patients with stage II and III CRC.

The overall study design is illustrated in Figure S1. During the discovery phase, we analysed the GSE54088 dataset^4^ to identify miRNAs differentially expressed in liver metastases compared to normal liver as well as normal colorectal mucosa. The heatmaps depicting the expression of seven candidate miRNAs are presented in Figure 1A, which clearly highlight that these candidates are unique and likely represent liver metastasis.

**FIGURE 1:**
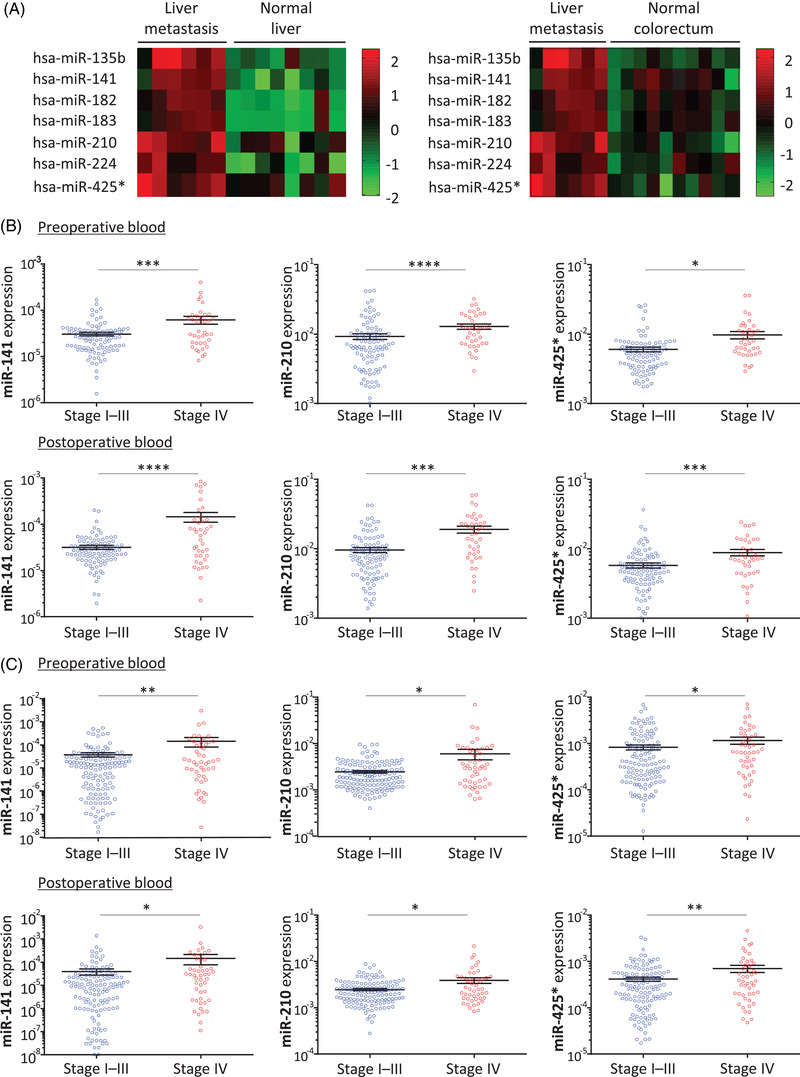
Identification of candidate metastasis‐associated miRNAs. (A) Heatmaps showing the differential expression (Z‐scores) of miRNAs in liver metastasis compared to surrounding normal liver tissues (left) and normal colorectal mucosae (right), based on miRNA microarray data from the GSE54088 dataset. (B,C) Circulating miR‐141, miR‐210, and miR‐425* expression levels in blood samples collected from (B) the cohort 1(*N* = 136) and (C) the cohort 2 (*N* = 180) before and after resection of primary CRC (stages I–III compared to stage IV). *P < .05; **P < .01, ***P < .001, ****P < .0001

Next, we obtained 25 formalin‐fixed, paraffin‐embedded metastatic CRC tissues (from the liver or lung), 22 matching normal liver or lung tissues, and 6 matching normal colorectal mucosae to examine the differential expression of the candidate miRNAs using reverse transcription polymerase chain reaction (qRT‐PCR). It was quite re‐assuring to witness that all seven miRNAs were upregulated in liver or lung metastases tissues (Figure S2A,B).

In order to evaluate the clinical utility of candidate miRNAs as blood‐based biomarkers, we analysed blood specimens collected from two independent clinical cohorts before and after primary CRC resection. As a result, we identified three miRNAs (miR‐210, miR‐425* and miR‐141) that were highly expressed in both pre‐ and post‐operative blood specimens from CRC patients with distant metastasis (stage IV) compared to patients without distant metastasis (stages I–III) in both cohorts (cohort 1 [*N *= 136]: Figure 1B, Figure S3A, cohort 2 [*N *= 180]: Figure 1C, Figure S3B). The high‐expression of these three miRNAs in stage IV CRC indicates that these tumour‐derived markers are likely shed into systemic circulation, making them attractive candidates for development as liquid‐biopsy markers for the identification of metastasis in patients with CRC. We analysed the associations between the expression levels of these miRNAs and key clinicopathological characteristics (Table S1: cohort 1, Table S2: cohort 2); indicating that our candidates are more related to haematogenous metastasis instead of lymphatic metastasis in CRC patients. In other words, we speculate that these miRNAs might be shed from CRC cells through venous invasion and could be detectable in blood.

To further investigate whether the circulating metastasis‐associated miRNAs could be used to predict prognosis in patients with CRC, we next analysed the OS of all CRC patients in our cohorts (stages I–IV) and the disease‐free survival (DFS) of the patients with stages II and III CRC. Kaplan–Meier analyses revealed that patients with high preoperative or postoperative miR‐210 and miR‐425*, or high postoperative miR‐141 levels exhibited significantly worse OS in both clinical cohorts (Figure S4A: cohort 1, Figure S4B: cohort 2). Kaplan–Meier analyses revealed that patients with high preoperative miR‐210 or postoperative miR‐425* levels had significantly worse DFS in both cohorts (Figure 2A: cohort 1, Figure 2D: cohort 2). We performed univariate Cox proportional hazards model analyses for predicting the DFS in the cohort 1 and 2 (Figure 2B,E) followed by multivariate analysis (Figure 2C,F). Multivariate analysis revealed preoperative miR‐210 expression and postoperative miR‐425* expression were independent factors for predicting poor DFS in both the cohort 1 (hazard ratio [HR]: 2.33, 95% confidence interval [CI]: 1.01–5.37, *P *= .04 and HR: 2.48, 95% CI: 1.13–5.43, *P* = .02, respectively;) and the cohort 2 (HR: 3.03, 95% CI: 1.04–8.87, *P* = .04 and HR: 5.82, 95% CI: 2.10–16.09, *P *< 0.001, respectively). Taken together, these data support the prognostic significance of both preoperative miR‐210 expression and early postoperative miR‐425* expression as blood‐based biomarkers in multiple clinical cohorts of patients with stage II and III CRC.

**FIGURE 2:**
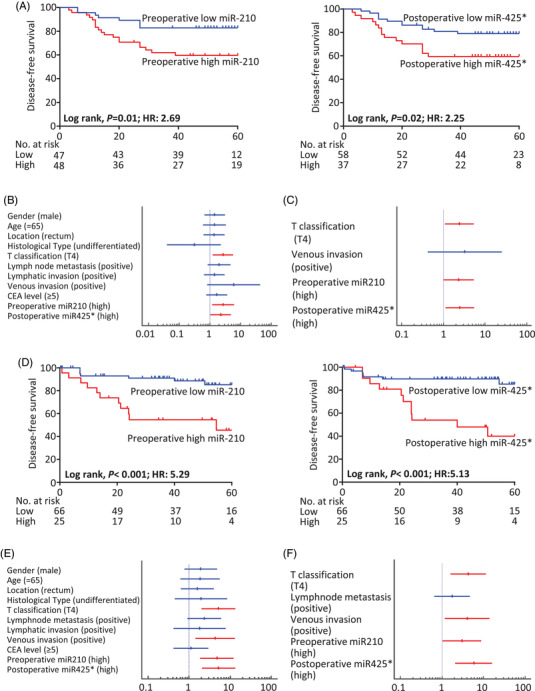
Disease‐free survival analysis with the candidate miRNAs in CRC patients. Kaplan–Meier survival plots for DFS of patients with stage II and III CRC, stratified by expression levels of circulating preoperative miR‐210 and postoperative miR‐425*, from (A) the cohort 1 and (D) the cohort 2. Hazard ratios of each clinicopathological factor and the expression of the two circulating miRNAs for predicting DFS in patients with stage II and III CRC, based on (B) univariate analysis in the cohort 1, (C) multivariate analysis in the cohort 1, (E) univariate analysis in the cohort 2, and (F) multivariate analysis in the cohort 2

Next, we constructed DFS prediction models for patients with stage II and III CRC in the cohort 2, by evaluating the predictive potential of the combination of circulating preoperative miR‐210 and postoperative miR‐425* using Cox proportional hazard models. This combination yielded an area under the time‐dependent receiver operating characteristic (ROC) curve (AUC) of 0.795 (Figure 3A). This model could also robustly predict DFS (HR: 9.10, 95% CI 2.47–33.48; *P *< .001). Thereafter, we constructed a new DFS prediction model combining the two‐circulating metastasis‐associated miRNAs with these two clinicopathological variables (T4 classification and venous invasion positivity), which yielded an AUC of 0.859 (Figure 3A). This model predicted DFS even more efficiently (HR: 12.02, 95% CI: 3.59–40.22; *P *< .001; Figure 3B). In addition, we evaluated this DFS prediction model in patients with stage II and III CRC separately, demonstrating that high scores were associated with lower DFS rates for patients in both groups (*P* < .001 for both; Figure 3C,D). Collectively, these results demonstrate the clinical significance of our circulating miRNA combination for risk stratification in patients with stage II and III CRC, as well as stage II CRC for whom actionable biomarkers are urgently needed in the clinic for the identification of optimal subgroup of patients who can benefit from adjuvant chemotherapy.

**FIGURE 3:**
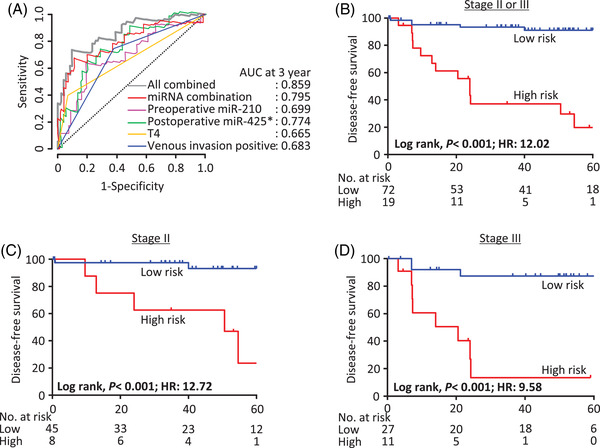
Risk‐stratification of colorectal cancer (CRC) patients based upon expression of miRNAs. (A) Time‐dependent receiver operating characteristic (ROC) curves comparing the accuracy of 3‐year disease‐free survival (DFS) predictions in the cohort 2 using models incorporating expression levels of circulating preoperative miR‐210 and postoperative miR‐425*, T4 classification status, venous invasion status, or all combined. Kaplan–Meier survival plots for DFS of patients, stratified by the combination model as high‐ or low‐risk, with (B) stage II or III, (C) stage II, and (D) stage III CRC

Several investigators have identified and demonstrated the clinical significance of metastasis‐specific tissue‐based biomarkers that were upregulated in metastases compared to primary CRC.^5^, ^6^ However, high reliance on such comparisons might have led to missing significant candidate miRNA biomarkers that are upregulated in both metastasis and primary CRC compared to normal tissue. Therefore, in the in silico miRNA discovery phase of this study, we aimed to identify candidate miRNAs that were upregulated in liver metastasis compared to normal colon mucosa and surrounding normal liver, ensuring that we can successfully develop clinically actionable circulating metastasis‐associated miRNAs.

Our study has potential limitations, given its retrospective nature. We did not have access to the status of microsatellite instability, which might be relevant additional clinical information for treatment selection for adjuvant therapy. Therefore, our results must be validated in a prospective, multi‐centre clinical trial, to evaluate the potential of this circulating miRNA combination for recurrence prediction.

In conclusion, we provide novel and clinically important evidence that our circulating metastasis‐associated miRNA signature which can be detected in pre‐operative and early post‐operative blood samples, can effectively risk‐stratify patients with stage II and III CRC. These circulating miRNAs offer tremendous clinical potential for directing personalised treatment regimens and the clinical management of patients with this lethal malignancy.

## CONFLICT OF INTEREST

The authors declare that there is no conflict of interest that could be perceived as prejudicing the impartiality of the research reported.

## FUNDING

The National Cancer Institute, National Institutes of Health (CA72851, CA181572, CA184792, CA187956) to A. Goel

## Supporting information

Supporting information
