# Supplementary material for: A metastasis‐associated microRNA‐based liquid biopsy signature for risk‐stratification in colorectal cancer: a multicenter cohort study
Source: Clin Transl Med. 2022 Dec 13;12(12):e998. doi: 10.1002/ctm2.998 (PMC9747679; doi:10.1002/ctm2.998)
Supplement: Supplementary file 1 — Supporting information [file CTM2-12-e998-s001.docx]

**A metastasis-associated microRNA-based liquid biopsy signature for risk-stratificaiton in colorectal cancer: A multicenter cohort study**

Takatoshi Matsuyama^1,4^, Yuji Toiyama^2^, Toshiaki Ishikawa^3^, Yoshinaga Okugawa^2^, Masamichi Yasuno^4^, Joan Maurel^5^, Yusuke Kinugasa^4^, Hiroyuki Uetake^3^, and Ajay Goel^1,6,7^

^1^ Center for Gastrointestinal Research, Center for Translational Genomics and Oncology, Baylor Scott & White Research Institute and Charles A Sammons Cancer Center, Baylor University Medical Center, Dallas, TX, USA.

^2^ Department of Gastrointestinal and Pediatric Surgery, Division of Reparative Medicine, Institute of Life Sciences, Graduate School of Medicine, Mie University, Mie, Japan.

^3^ Department of Specialized Surgery, Tokyo Medical and Dental University Graduate School of Medicine, Tokyo, Japan.

^4^ Department of Gastrointestinal Surgery, Tokyo Medical and Dental University Graduate School of Medicine, Tokyo, Japan.

^5^ Translational Genomics and Targeted Therapeutics in Solid Tumors Group Medical Oncology, Hospital Clinic of Barcelona, CIBERehd, IDIBAPS, Barcelona, Spain

^6^ Department of Molecular Diagnostics and Experimental Therapeutics, Beckman Research Institute of City of Hope, Monrovia, CA, USA

^7^ City of Hope Comprehensive Cancer Center, Duarte, CA, USA.

**Supplementary data**

**Figure S1:** The study design for the identification and validation of the circulating metastasis-associated miRNAs for CRC

**
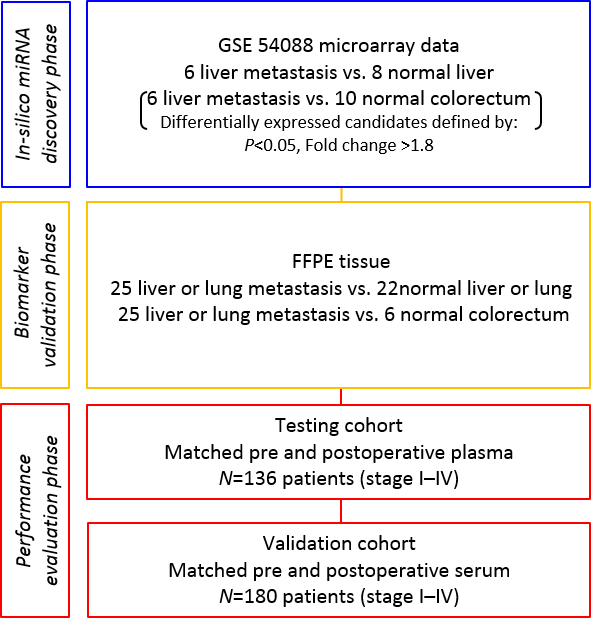
**

**Figure S2:** Tissue validation of seven candidate metastasis-associated miRNAs in FFPE samples. liver or lung metastasis (metastasis) was compared to B) surrounding matched normal tissue (MN) around metastasis site or C) matching normal colorectal (NC) mucosae of an independent cohort of patients with CRC. *P < 0.05; **P< 0.01, ***P< 0.001, ****P<0.0001.

**Figure S3:** Difference of candidate circulating miRNAs’ expressions between stage IV patients and stage I–III patients. (A) cohort 1, (B) cohort 2. *P < 0.05; **P< 0.01, ****P<0.0001, N.S.: not significant.


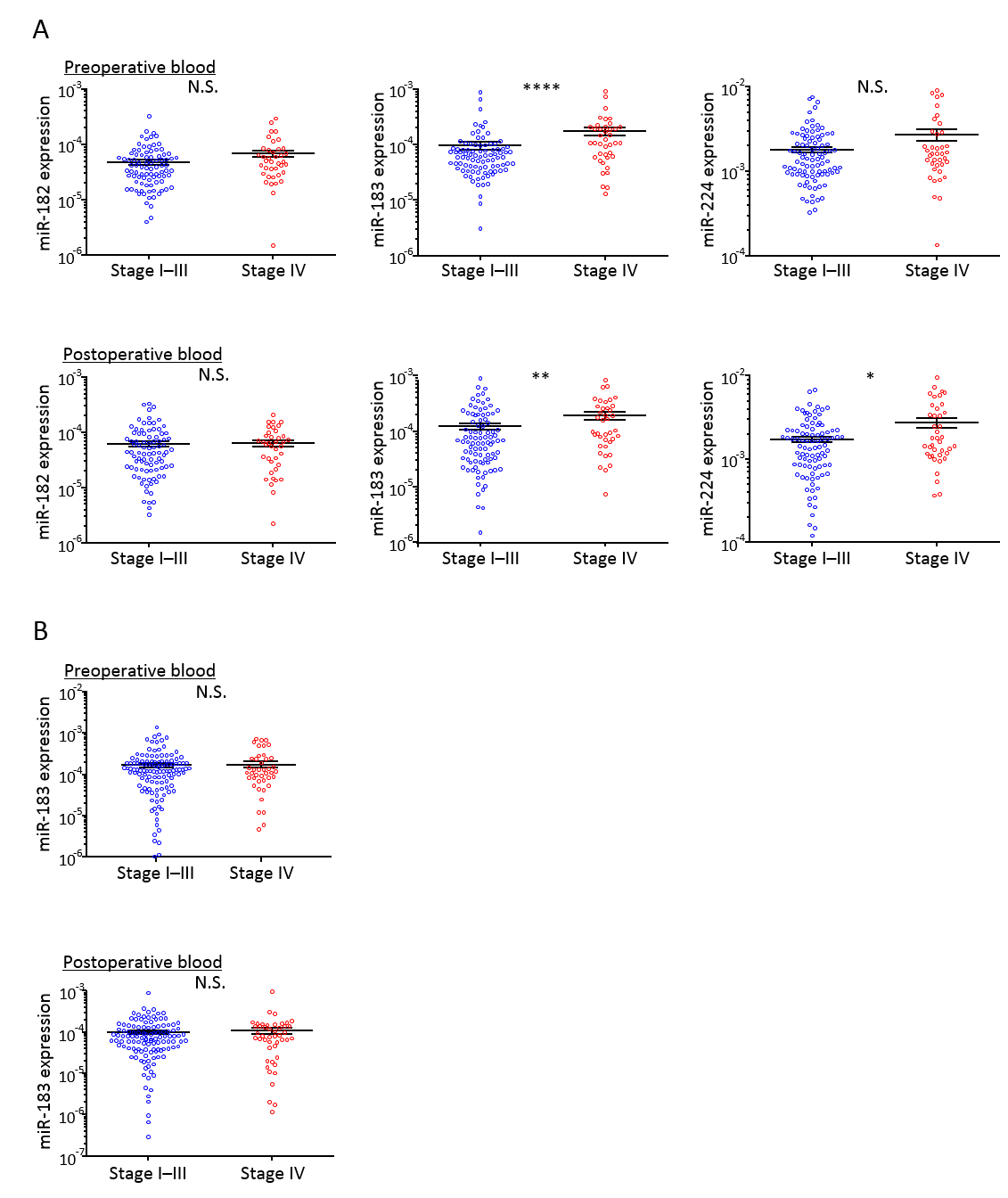


**Figure S4:** Over-all Survival analysis with the candidate miRNAs in CRC patients. Kaplan–Meier survival plots for OS of patients with CRC, stratified by circulating miR-210, miR-425*, or miR-141 expression levels in (A) cohort 2, (B) cohort 2.
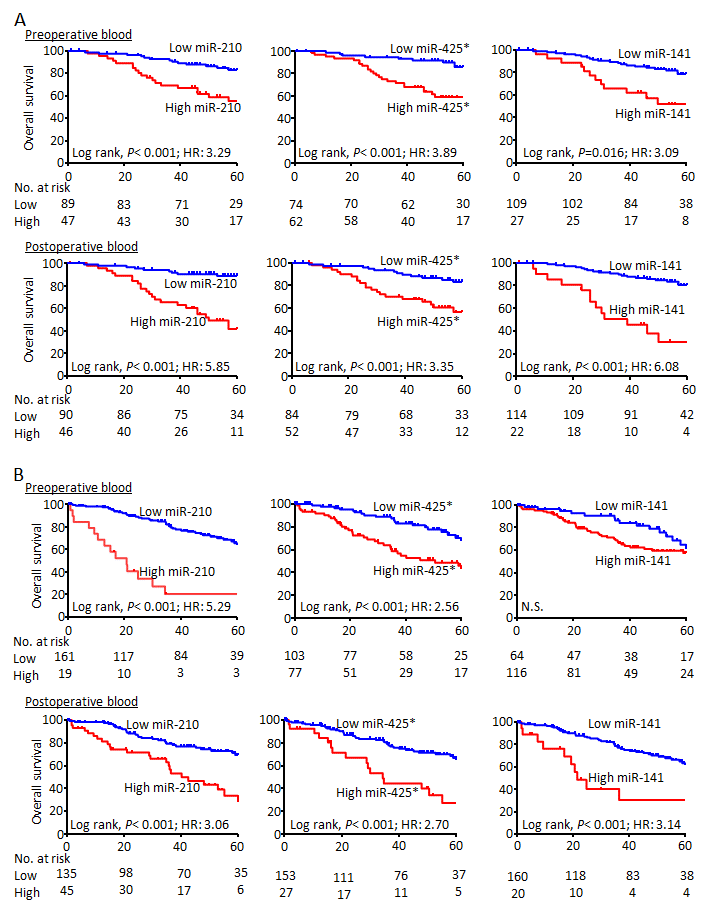


**Table S1.** Associations between the expression of circulating miRNAs in pre-operative blood specimens and clinicopathological factors in the cohort 1

|  | **miR-141** | | | **miR-210** | | | **miR-425*** | | | **Postoperative miR-141** | | | **Postoperative miR-210** | | | **Postoperative miR-425*** | | |
| --- | --- | --- | --- | --- | --- | --- | --- | --- | --- | --- | --- | --- | --- | --- | --- | --- | --- | --- |
|  | Low | High |  | Low | High |  | Low | High |  | Low | High |  | Low | High |  | Low | High |  |
| **Variables** | *N*=109 | *N*=27 | *P* value | *N*=89 | *N*=47 | *P* value | *N*=74 | *N*=62 | *P* value | *N*=114 | *N*=22 | *P* value | *N*=90 | *N*=46 | *P* value | *N*=84 | *N*=52 | *P* value |
| **Gender** |  |  |  |  |  |  |  |  |  |  |  |  |  |  |  |  |  |  |
| Male | 53 | 16 |  | 49 | 20 |  | 32 | 37 |  | 58 | 11 |  | 47 | 22 |  | 35 | 34 |  |
| Female | 56 | 11 | 0.32 | 40 | 27 | 0.16 | 42 | 25 | 0.05 | 56 | 11 | 0.93 | 43 | 24 | 0.62 | 49 | 18 | **0.007** |
| **Age** |  |  |  |  |  |  |  |  |  |  |  |  |  |  |  |  |  |  |
| < 65 | 37 | 15 |  | 36 | 16 |  | 23 | 29 |  | 42 | 10 |  | 31 | 21 |  | 21 | 31 |  |
| ≥ 65 | 72 | 12 | **0.03** | 53 | 31 | 0.46 | 51 | 33 | 0.06 | 72 | 12 | 0.44 | 59 | 25 | 0.20 | 63 | 21 | **< 0.001** |
| **Location** |  |  |  |  |  |  |  |  |  |  |  |  |  |  |  |  |  |  |
| Colon | 78 | 16 |  | 59 | 35 |  | 52 | 42 |  | 79 | 15 |  | 64 | 30 |  | 65 | 29 |  |
| Rectum | 31 | 11 | 0.21 | 30 | 12 | 0.32 | 22 | 20 | 0.75 | 35 | 7 | 0.91 | 26 | 16 | 0.48 | 19 | 23 | **0.008** |
| **Histology** |  |  |  |  |  |  |  |  |  |  |  |  |  |  |  |  |  |  |
| Differentiated | 94 | 27 |  | 81 | 40 |  | 69 | 52 |  | 101 | 20 |  | 81 | 40 |  | 75 | 46 |  |
| Undifferentiated | 14 | 0 | **0.04** | 8 | 6 | 0.46 | 5 | 9 | 0.12 | 12 | 2 | 0.82 | 9 | 5 | 0.84 | 9 | 5 | 0.86 |
| **T stage** |  |  |  |  |  |  |  |  |  |  |  |  |  |  |  |  |  |  |
| T1,T2 | 11 | 2 |  | 11 | 2 |  | 5 | 8 |  | 12 | 1 |  | 10 | 3 |  | 7 | 6 |  |
| T3,T4 | 98 | 25 | 0.67 | 78 | 45 | 0.12 | 69 | 54 | 0.22 | 102 | 21 | 0.38 | 80 | 43 | 0.38 | 77 | 46 | 0.53 |
| **Lymphatic invasion** | |  |  |  |  |  |  |  |  |  |  |  |  |  |  |  |  |  |
| Absent | 68 | 14 |  | 56 | 26 |  | 45 | 37 |  | 69 | 13 |  | 56 | 26 |  | 52 | 30 |  |
| Present | 41 | 13 | 0.31 | 33 | 21 | 0.38 | 29 | 25 | 0.89 | 45 | 9 | 0.89 | 34 | 20 | 0.52 | 32 | 22 | 0.62 |
| **Venous invasion** |  |  |  |  |  |  |  |  |  |  |  |  |  |  |  |  |  |  |
| Absent | 17 | 1 |  | 15 | 3 |  | 11 | 7 |  | 17 | 1 |  | 17 | 1 |  | 12 | 6 |  |
| Present | 92 | 26 | 0.10 | 74 | 44 | 0.08 | 63 | 55 | 0.54 | 97 | 21 | 0.18 | 73 | 45 | **0.006** | 72 | 46 | 0.64 |
| **Lymph node metastasis** | |  |  |  |  |  |  |  |  |  |  |  |  |  |  |  |  |  |
| Absent | 38 | 9 |  | 32 | 15 |  | 27 | 20 |  | 42 | 5 |  | 35 | 12 |  | 28 | 19 |  |
| Present | 71 | 18 | 0.88 | 57 | 32 | 0.63 | 47 | 42 | 0.60 | 72 | 17 | 0.20 | 55 | 34 | 0.13 | 56 | 33 | 0.70 |
| **Distant metastasis** | |  |  |  |  |  |  |  |  |  |  |  |  |  |  |  |  |  |
| Absent | 87 | 9 |  | 72 | 24 |  | 61 | 35 |  | 92 | 5 |  | 75 | 22 |  | 70 | 27 |  |
| Present | 22 | 18 | **< 0.001** | 17 | 23 | **< 0.001** | 13 | 26 | **0.001** | 22 | 17 | **< 0.001** | 15 | 24 | **< 0.001** | 14 | 25 | **< 0.001** |
| **Stage** |  |  |  |  |  |  |  |  |  |  |  |  |  |  |  |  |  |  |
| I, II | 34 | 5 |  | 27 | 12 |  | 24 | 15 |  | 36 | 3 |  | 30 | 9 |  | 27 | 12 |  |
| III, IV | 75 | 22 | 0.19 | 62 | 35 | 0.55 | 50 | 47 | 0.29 | 78 | 19 | 0.08 | 60 | 37 | 0.09 | 57 | 40 | 0.25 |
| **Preoperative CEA (ng/ml)** | |  |  |  |  |  |  |  |  |  |  |  |  |  |  |  |  |  |
| < 5 | 69 | 12 |  | 62 | 19 |  | 45 | 36 |  | 75 | 6 |  | 60 | 21 |  | 54 | 27 |  |
| ≥ 5 | 40 | 15 | 0.07 | 27 | 28 | **0.001** | 29 | 26 | 0.74 | 39 | 16 | **< 0.001** | 30 | 25 | **0.01** | 30 | 25 | 0.15 |

**Table S2.** Associations between the expression of circulating miRNAs in pre-operative blood specimens and clinicopathological factors in the cohort 2

|  | **miR-141** | | | **miR-210** | | | **miR-425*** | | | **Postoperative miR-141** | | | **Postoperative miR-210** | | | **Postoperative miR-425*** | | |
| --- | --- | --- | --- | --- | --- | --- | --- | --- | --- | --- | --- | --- | --- | --- | --- | --- | --- | --- |
|  | Low | High |  | Low | High |  | Low | High |  | Low | High |  | Low | High |  | Low | High |  |
| **Variables** | *N*=64 | *N*=116 | *P* value | *N*=161 | *N*=19 | *P* value | *N*=103 | *N*=77 | *P* value | *N*=160 | *N*=20 | *P* value | *N*=135 | *N*=45 | *P* value | *N*=153 | *N*=27 | *P* value |
| **Gender** |  |  |  |  |  |  |  |  |  |  |  |  |  |  |  |  |  |  |
| Male | 32 | 68 |  | 90 | 10 |  | 61 | 39 |  | 91 | 9 |  | 77 | 23 |  | 85 | 15 |  |
| Female | 32 | 48 | 0.26 | 71 | 9 | 0.78 | 42 | 38 | 0.25 | 69 | 11 | 0.31 | 58 | 22 | 0.48 | 68 | 12 | 0.99 |
| **Age** |  |  |  |  |  |  |  |  |  |  |  |  |  |  |  |  |  |  |
| < 65 | 19 | 48 |  | 60 | 7 |  | 38 | 29 |  | 57 | 10 |  | 44 | 23 |  | 55 | 12 |  |
| ≥ 65 | 45 | 68 | 0.12 | 101 | 12 | 0.97 | 65 | 48 | 0.91 | 103 | 10 | 0.20 | 91 | 22 | **0.02** | 98 | 15 | 0.39 |
| **Location** |  |  |  |  |  |  |  |  |  |  |  |  |  |  |  |  |  |  |
| Colon | 36 | 68 |  | 93 | 11 |  | 59 | 45 |  | 90 | 14 |  | 76 | 28 |  | 88 | 16 |  |
| Rectum | 28 | 48 | 0.75 | 68 | 8 | 0.99 | 44 | 32 | 0.87 | 70 | 6 | 0.24 | 59 | 17 | 0.48 | 65 | 11 | 0.86 |
| **Histology** |  |  |  |  |  |  |  |  |  |  |  |  |  |  |  |  |  |  |
| Differentiated | 61 | 103 |  | 152 | 12 |  | 98 | 66 |  | 149 | 15 |  | 123 | 41 |  | 143 | 11 |  |
| Undifferentiated | 3 | 12 | 0.18 | 9 | 6 | **<0.001** | 5 | 10 | **0.04** | 10 | 5 | **0.004** | 11 | 4 | 0.88 | 10 | 5 | **<0.001** |
| **T stage** |  |  |  |  |  |  |  |  |  |  |  |  |  |  |  |  |  |  |
| T1,T2 | 23 | 30 |  | 50 | 3 |  | 35 | 18 |  | 47 | 6 |  | 46 | 7 |  | 47 | 6 |  |
| T3,T4 | 41 | 85 | 0.16 | 111 | 15 | 0.20 | 68 | 58 | 0.13 | 112 | 14 | 0.96 | 88 | 38 | **0.01** | 106 | 20 | 0.43 |
| **Lymphatic invasion** | |  |  |  |  |  |  |  |  |  |  |  |  |  |  |  |  |  |
| Absent | 16 | 26 |  | 42 | 0 |  | 26 | 16 |  | 35 | 7 |  | 33 | 9 |  | 37 | 5 |  |
| Present | 48 | 89 | 0.71 | 119 | 18 | **0.013** | 77 | 60 | 0.51 | 124 | 13 | 0.19 | 101 | 36 | 0.52 | 116 | 21 | 0.58 |
| **Venous invasion** |  |  |  |  |  |  |  |  |  |  |  |  |  |  |  |  |  |  |
| Absent | 39 | 60 |  | 95 | 4 |  | 66 | 33 |  | 92 | 7 |  | 78 | 21 |  | 88 | 11 |  |
| Present | 25 | 55 | 0.25 | 66 | 14 | **0.003** | 37 | 43 | **0.006** | 67 | 13 | 0.05 | 56 | 24 | 0.17 | 65 | 15 | 0.14 |
| **Lymph node metastasis** | |  |  |  |  |  |  |  |  |  |  |  |  |  |  |  |  |  |
| Absent | 39 | 60 |  | 96 | 3 |  | 62 | 16 |  | 91 | 8 |  | 82 | 17 |  | 89 | 10 |  |
| Present | 25 | 55 | 0.25 | 65 | 15 | **<0.001** | 41 | 60 | **<0.001** | 68 | 12 | 0.14 | 52 | 28 | **0.006** | 64 | 16 | 0.06 |
| **Distant metastasis** | |  |  |  |  |  |  |  |  |  |  |  |  |  |  |  |  |  |
| Absent | 48 | 80 |  | 127 | 5 |  | 83 | 49 |  | 123 | 9 |  | 107 | 25 |  | 117 | 15 |  |
| Present | 12 | 36 | 0.11 | 34 | 14 | **<0.001** | 20 | 28 | **0.01** | 37 | 11 | **0.002** | 28 | 20 | **0.002** | 36 | 12 | **0.02** |
| **Stage** |  |  |  |  |  |  |  |  |  |  |  |  |  |  |  |  |  |  |
| I, II | 38 | 57 |  | 93 | 2 |  | 61 | 34 |  | 88 | 7 |  | 78 | 17 |  | 86 | 9 |  |
| III, IV | 26 | 59 | 0.18 | 68 | 17 | **<0.001** | 42 | 43 | **0.04** | 72 | 13 | 0.09 | 57 | 28 | **0.01** | 67 | 18 | **0.02** |
| **Preoperative CEA (ng/ml)** | |  |  |  |  |  |  |  |  |  |  |  |  |  |  |  |  |  |
| < 5 | 30 | 42 |  | 69 | 3 |  | 44 | 28 |  | 66 | 6 |  | 56 | 16 |  | 65 | 7 |  |
| ≥ 5 | 32 | 71 | 0.14 | 88 | 15 | **0.02** | 57 | 46 | 0.44 | 89 | 14 | 0.28 | 75 | 28 | 0.45 | 86 | 17 | 0.19 |
